# Validation of a Wearable Medical Device for Automatic Diagnosis of OSA against Standard PSG

**DOI:** 10.3390/jcm13020571

**Published:** 2024-01-19

**Authors:** Jesus Sanchez Gomez, Renard Xaviero Adhi Pramono, Syed Anas Imtiaz, Esther Rodriguez-Villegas, Agustin Valido Morales

**Affiliations:** 1 Sleep Unit, Pneumology Department, Virgen Macarena University Hospital, 41009 Seville, Spain; jesus.charca.sspa@juntadeandalucia.es (J.S.G.); agustins.valido.sspa@juntadeandalucia.es (A.V.M.); 2 Wearable Technologies Lab, EEE Department, Imperial College London, London SW7 2AZ, UK; anas.imtiaz@imperial.ac.uk (S.A.I.); e.rodriguez@imperial.ac.uk (E.R.-V.)

**Keywords:** OSA, sleep, sleep apnea, hsat, PSG, wearable, sleep testing, AHI, ODI

## Abstract

Study objective: The objective of this study was to assess the accuracy of automatic diagnosis of obstructive sleep apnea (OSA) with a new, small, acoustic-based, wearable technology (AcuPebble SA100), by comparing it with standard type 1 polysomnography (PSG) diagnosis. Material and methods: This observational, prospective study was carried out in a Spanish hospital sleep apnea center. Consecutive subjects who had been referred to the hospital following primary care suspicion of OSA were recruited and underwent in-laboratory attended PSG, together with the AcuPebble SA100 device simultaneously overnight from January to December 2022. Results: A total of 80 patients were recruited for the trial. The patients had a median Epworth scoring of 10, a mean of 10.4, and a range of 0–24. The mean AHI obtained with PSG plus sleep clinician marking was 23.2, median 14.3 and range 0–108. The study demonstrated a diagnostic accuracy (based on AHI) of 95.24%, sensitivity of 92.86%, specificity of 97.14%, positive predictive value of 96.30%, negative predictive value of 94.44%, positive likelihood ratio of 32.50 and negative likelihood ratio of 0.07. Conclusions: The AcuPebble SA100 (EU) device has demonstrated an accurate automated diagnosis of OSA in patients undergoing in-clinic sleep testing when compared against the gold-standard reference of in-clinic PSG.

## 1. Introduction

Obstructive sleep apnea (OSA) is a common sleep disorder characterized by repetitive pauses in breathing during sleep, resulting in disrupted sleep patterns and potential health complications. It is estimated that OSA affects over 936 million adults aged 30–69 years (men and women), out of whom 425 million have moderate to severe severity [1]. Unfortunately, the vast majority of those are not diagnosed and the disease is only identified once co-morbidities have been developed [2]. One of the main issues faced by individuals with OSA is excessive daytime sleepiness, which can affect their productivity, concentration, and overall quality of life. It is estimated that people with OSA have between 3 to 6 greater risk of suffering motor vehicle accidents [3]. Moreover, OSA has been linked to an increased risk of developing other health conditions, such as high blood pressure, ischemic heart disease, and dementia [4,5,6,7]. And untreated OSA is associated with an increased risk of all-cause and cardiovascular mortality [8]. Aside from the physical and emotional toll that obstructive sleep apnea takes on individuals, it also carries a significant financial burden; the costs, both direct and indirect, have been demonstrated to be huge [9]. All the risks, adverse outcomes, and overall financial burden of the disease can potentially be reduced with effective treatment [10]. Therefore, the diagnosis and effective treatment of OSA in adults is an urgent health priority [11].

Traditionally, OSA has been diagnosed through in-clinic polysomnography (PSG), which serves as the gold standard. This procedure involves a sleep study conducted in a healthcare laboratory setting, where a variety of physiological channels are measured. These generally include oximetry, snoring, body and leg position/movements, oro-nasal pressure and/or temperature as surrogates for nasal and/or mouth airflow corresponding to breathing, a respiratory effort that reflects on the movement of the chest and abdomen, electrocardiogram, electroencephalogram, electrooculogram, and electromyogram. These channels are then scored manually by expert sleep healthcare professionals who identify specific respiratory events, which are characteristic of the disease, and then combine the count in different indexes which determine whether the patient has got OSA, as well as the disease severity.

While PSG remains the gold standard for OSA diagnosis [12], undergoing the procedure can be both expensive and challenging: It requires the patient to spend the night in a healthcare facility; it limits normal sleep habits both because of the different environment as well as the burden imposed by the sensors (which can on its own affect diagnostic outputs); the equipment is bulky and expensive; and it requires a significant personnel time to set up and specialist time (up two hours) to analyze the signals and generate a diagnosis. Moreover, the process of manual scoring of the signals is subjective and error-prone, and this can influence the diagnosis [13]. All of these factors and limitations of PSG contribute towards making the diagnostic process far from ideal neither for patients, healthcare professionals, or healthcare bodies overall. Although in the last guidelines [12], there is a place for more simplified methods of diagnosis in a subgroup of patients, long waiting times until treatment are frequent [14] and a very large percentage of patients remain undiagnosed [9].

To address some of the challenges and limitations above, alternative diagnostic methods and devices for OSA have emerged. One such medical device is AcuPebble SA100 (EU), which has been granted regulatory approval for fully automated diagnosis of OSA in both hospital and home environments. This device is small, wearable, very easy to use, low-cost, and amongst other things, it also addresses some of the issues associated with diagnosis in specific populations, increasing diagnostic accessibility. The proprietary device core operation is based on detecting a mixture of acoustic signals generated by different physiological functions, algorithmically separating those signals into channels, and also automatically extracting events and information that is relevant for the diagnosis of OSA. Previous studies have already validated its performance and demonstrated its efficacy for diagnosing OSA in comparison to home-based polygraphy testing [15]. The objective of this study is to further validate the effectiveness of the AcuPebble SA100 device in a clinical setting by comparing its diagnostic results with those obtained from PSG.

## 2. Materials and Methods

### 2.1. Study Protocol

The trial, which was an observational, prospective, blinded, diagnostic accuracy study, was designed in adherence to the ISO Standard on Clinical Investigation of Medical Devices for Human Subjects EN ISO 14155:2011 [16], as well as the recommendations of the *British Medical Journal* on assessments and critical appraisals of diagnostic tests [17] and the USA Agency for Healthcare Research and Quality for studies specific to diagnosis and treatment of sleep apnea in adults [18]. The study was approved by the ethics committees of the University Hospitals Vírgen Macarena-Virgen del Rocío and also by the Spanish Agency of Medicines and Medical Devices (AEMPS, ID: 720/19/EC) and was carried out in the Sleep Unit of the Respiratory Service at the Virgen Macarena University Hospital in Seville, Spain. The trial registration number was NCT04028011, and the primary endpoint was to compare the automatic diagnostic results of AcuPebble SA100 with those obtained from PSG in terms of sensitivity and specificity. The secondary endpoints of the trial, within the scope of this paper, were to also obtain the positive and negative predictive values, likelihood ratios, and classification of different types of apnea events. Patients who had been referred for assessment of potential OSA, following the conventional pathway referral process in the Andalucia region of Spain—which also followed the Spanish pathways for OSA diagnosis guidelines—were recruited consecutively between January 2022 and December 2022.

**Figure 1:**
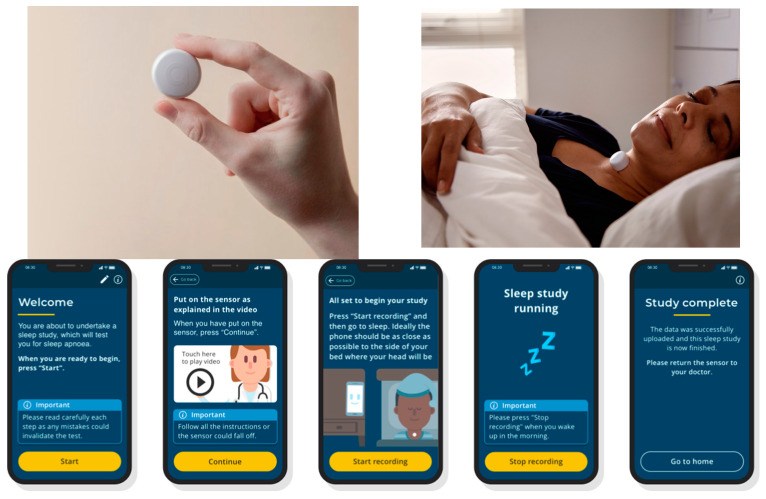
(**Top**-**left**) AcuPebble sensor; (**Top**-**right**) a model wearing the AcuPebble sensor; (**Bottom**) screenshots of the AcuPebble SA100 mobile application.

Patients were given details in advance about the study, both verbally and in writing. They were also asked to sign an informed consent on the day of their study if they chose to participate. On the day of their sleep study, patients were first set up with the complete suite of conventional PSG sensors. Following this, they were given an AcuPebble sensor, a mobile phone, and a basic instructions sheet explaining how to open the AcuPebble SA100 application on the mobile phone (i.e., turn on the mobile phone and tap the application icon). Further instructions are part of the mobile application. The patient is guided using a series of screens and videos enabling them to understand the test and how to put on the AcuPebble sensor, so that they can start the test independently themselves. Figure 1 shows the AcuPebble sensor, a model wearing the sensor, as well as some screenshots of the application.

### 2.2. Eligibility Criteria

The trial included consecutive adult patients aged 18 to 80 who were referred to the sleep clinic for possible sleep apnea diagnosis. Exclusion criteria included non-Spanish speakers or individuals with special communication needs preventing use of the mobile–app interface of AcuPebble SA100, those with physical or mental impairments preventing unassisted use of the technology, known allergy to adhesive dressings used to attach AcuPebble SA100 sensor, and excessive loose skin in the neck area as that would invariably result in the AcuPebble device swinging with slight movement of the neck.

### 2.3. Reference Standard

The reference diagnostic standard for this trial was the manual scoring of the in-clinic polysomnography output. The PSG system used was the Philips Sleepware G3 version 2.8.78 (Eindhoven, The Netherlands). The signals recorded were electroencephalogram, electrooculogram, chin electromyogram, respiratory airflow (measured using naso-oral thermistor and nasal pressure airflow signals), chest and abdominal wall movements (by thoracic and abdominal piezo belts), oxygen saturation (by finger oximeter), anterior tibialis electromyography, lead II electrocardiography, snoring, and body position. In addition, a video camera equipped with infrared LEDs recorded each subject while sleeping. Blind manual scoring was carried out by a trained sleep physician following the 2017 American Academy of Sleep Medicine (AASM) guidelines [19] as the criteria, defining apnea as a reduction of nasal flow by at least 90% of baseline for at least 10 s and hypopnea as a reduction of nasal flow by at least 30% of baseline for at least 10 s accompanied by SpO2 decrease of at least 3% and/or arousal. The AHI was calculated as the total number of apneas and hypopneas divided by the total number of hours of sleep determined by PSG. PSG results were considered of sufficient quality if a recording of at least 240 min of total sleep time was obtained.

### 2.4. AcuPebble SA100

The device, whose automatic output was compared to that of the conventional PSG manually scored gold standard, was the European variant of AcuPebble SA100 (Figure 1). This, which was previously described in [15], consists of a small wearable sensor, algorithms that are able to separate physiological channels, extract clinical parameters of interest and also a fully automatic diagnosis, and a user-friendly mobile application that provides simple instructions to patients. These instructions involve peeling off the adhesive on the back of the device and attaching it to the front of the neck. Patients are also instructed to place their mobile phones within 1 m of their bed. They can initiate the test when going to sleep and stop it upon waking up by tapping a button on their mobile phone. The collected data are subsequently uploaded to AcuPebble SA100′s cloud platform, where it undergoes analysis utilizing the proprietary software algorithms. This analysis generates a diagnostic output in accordance with the recommendations provided by the AASM.

### 2.5. Data Analysis

The main objective of this study was to compare the automatic diagnostic output of AcuPebble SA100 against the gold standard in-clinic polysomnography. More specifically, PSG signals were manually scored by the clinicians, and patients were classified as having mild, moderate, or severe OSA, as per the 5, 15, and 30 indices thresholds [19], respectively, taking into account two different criteria:

- **AHI criteria**: Diagnosis based on AHI, marking apnea and hypopnea as defined by the current recommended AASM rules, i.e., for apnea, there is a drop in the peak signal excursion by ≥90% of pre-event baseline. For hypopnea, there is a drop in the peak signal excursion by ≥30% of the pre-event baseline, and there is a ≥3% oxygen desaturation from the pre-event baseline or the event is associated with an arousal. The duration of the drop in signal excursion is ≥10 s for both apnea and hypopnea.
- **ODI criteria**: Diagnosis based on ODI, marking desaturation events as a drop of *≥*3% in the oxygen saturation value.

Clinicians did this blindly and at no point had any information about the automatic diagnosis produced by AcuPebble SA100. Sensitivity, specificity, accuracy, positive and negative predictive values, and positive and negative likelihood ratios were used as statistical metrics for comparison. Medcalc software was used to compute the metrics (URL: https://www.medcalc.org/, accessed on 12 November 2023), where the 95% confidence intervals were also calculated.

The evaluation of diagnostic performance was based on the following criteria:

- A diagnostic output was classified as a *True Positive (TP)* when both AcuPebble SA100 and the gold standard concurred on diagnosing a patient with either moderate or severe sleep apnea, that is, when the threshold for the index in comparison is 15 events/h. This classification was chosen because moderate and severe OSAs are the diagnostic categories for which gold-standard treatment (continuous positive airway pressure (CPAP)) is recommended [20].
- A diagnostic output was labeled as a *True Negative (TN)* when both AcuPebble SA100 and the gold standard agreed that the patient did not have moderate or severe OSA.
- A diagnostic output from AcuPebble SA100 was deemed a *False Negative (FN)* if the gold standard diagnosed the patient with moderate or severe OSA, while the AcuPebble SA100 diagnosis indicated mild or normal.
- A diagnostic output from AcuPebble SA100 was identified as a *False Positive (FP)* if the gold standard diagnosed the patient with normal or mild sleep apnea, and the AcuPebble SA100 diagnosis suggested moderate or severe sleep apnea.

### 2.6. Sample Calculation

The results reported here are the first part of a two-part study; the second part comprises patients who were being diagnosed whilst using cardio-respiratory polygraphy at home. The original sample calculation considered both parts simultaneously.

The number of patients in that sample was chosen according to the recommendations of the Agency for Healthcare Research and Quality (AHRQ, Rockville, MD, USA) for the diagnosis and treatment of obstructive sleep apnea in a hospital. These recommendations and their statistical methods are in accordance with the methods published by the *British Medical Journal*, BMJ [17], and the recommendations of Flemons et al. [21] regarding the statistical validation of ambulatory monitors for the diagnosis of sleep apnea. Based on these recommendations, a sample of 150 was suitable because, according to clinical knowledge, it is expected that the prevalence of the disease among patients referred for diagnosis would be of the order of 50%. Assuming a sensitivity of 90% and a specificity of 99%, with this sample the positive probability ratio would be 90 (the value recommended by the literature mentioned above is more than 10), which would result in a post-test probability of 99% (that is, approximately 1 in 1 patient who test positive have sleep apnea), with 95% confidence intervals between 90% and 100%. The negative likelihood ratio would be 0.1 (also within the recommendation), resulting in a posterior probability of 9% with 95% confidence intervals between 5% and 17%. These results can be reproduced using the University of Illinois Chicago online calculator [22]. Furthermore, with this sample number, the 95% confidence intervals for sensitivity and specificity will be (82%, 96%) and (93%, 100%).

Although the second part of the study did not take place, the reason was that an identical study with an equivalent population had already finished [15]; hence, data corresponding to this sample were already available. It is also for this reason that an amendment to this second part of the study was approved by both the local ethics committee and the Spanish Agency of Medicine and Medical Device to evaluate a different endpoint.

## 3. Results

### 3.1. Participants

**Figure 2:**
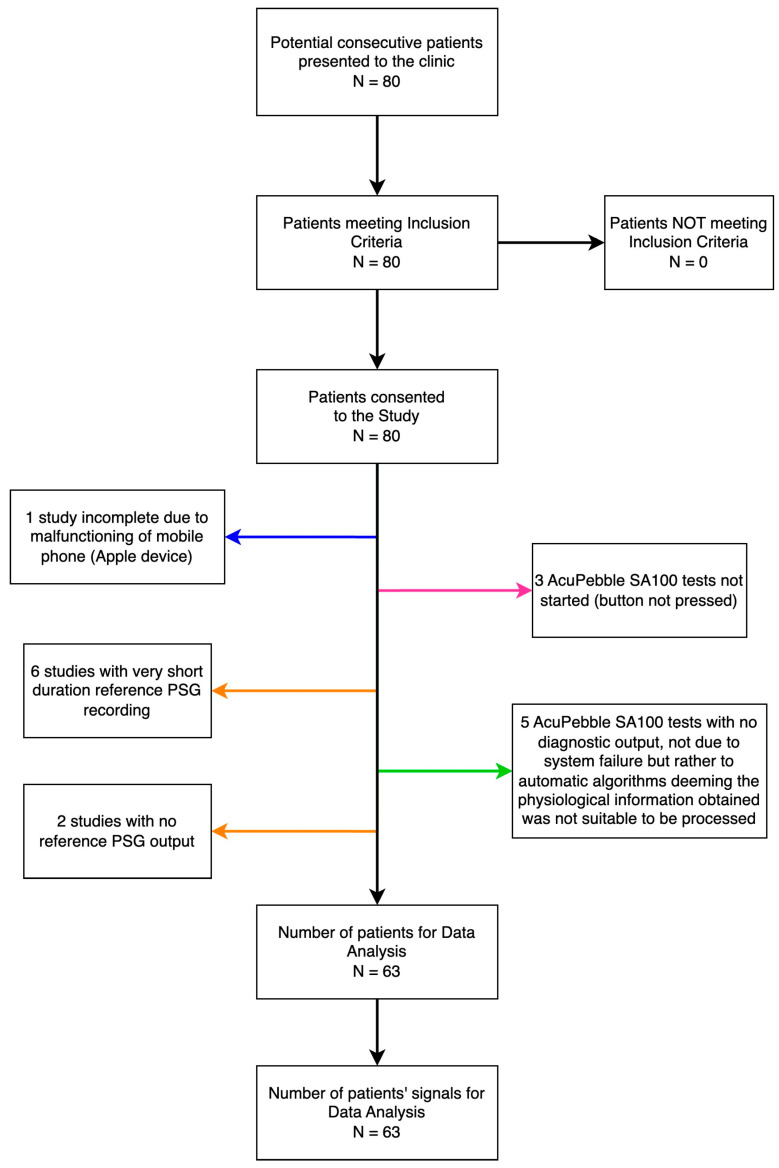
The flow of participants and data sufficiency. Arrows in orange point at no data for comparison due to the reference standard, in blue to a third-party accessory, in pink to not compliance with the protocol, and in green as a result of no diagnostic output of AcuPebble SA100, not due to system failure but rather to automatic algorithms deeming the physiological information obtained from the sensed signal was not suitable to be processable (Note: This can happen for a variety of reasons, including the algorithms detecting the signals indicate the instructions for use have not been followed, wrong sensor location, patient likely not in bed, etc.).

A total of 80 consecutive participants meeting the eligibility criteria were consecutively enrolled in this prospective study. The trial took place at the Virgen Macarena Hospital between January 2022 and December 2022. Out of the total, 63 studies could be used for comparison. The 17 remaining patients could not be included due to 1 third-party equipment malfunction only observed post-recruitment, 6 short duration of reference PSG recording, 2 due to lack of output from the reference recording due to software-related failure in the computer running the reference standard system (of these one of the patients had also been annotated in the CRF not to have slept at all), 3 due to human error in the form of forgetting to start the AcuPebble SA100 test, and 5 due to the algorithms of AcuPebble SA100 not producing any diagnosis, but rather an output determining there was lack of confidence from the signals to produce this. (This is a safety-by-design feature of the system to minimize the risk of a wrong diagnosis, which can trigger for a variety of reasons, such as signals not representative of intended use as per the user manual, signals indicative of instructions of use not followed, etc.). A data sufficiency diagram is shown in Figure 2.

Among the participants, 63% were men (40 males and 23 females). They had a median age of 48 years, ranging between a minimum age of 25 years and a maximum of 78 years. Table 1 presents detailed demographic characteristics data for the patients, including BMI classifications and sleepiness scores using Epworth and Stop Bang questionnaires. Additionally, a number of comorbidities were reported among the participants. These include hypertension, diabetes, cardiopathies, nocturia, turbinoplasty, hypothyroidism, floppy eyelid syndrome, depressive anxiety disorder, dyslipidemia, irritable colon, megaloblastic anemia, lumbociatalgia, sleep onset insomnia, asthma, allergic rhinitis, sleep maintenance insomnia, multiple sclerosis, posterior territory ischemic stroke (POCI) of probable mesencephalic–protuberancial localization, HIV, bronchial hyper-reactivity, depressive disorder, Crohn’s disease, epilepsy, turbinate hypertrophy, chronic diarrhea, Gilbert’s syndrome, scoliosis, fibromyalgia, bruxism, sleepwalking, diabetic neuropathy, multinodular goiter, psoriatic arthritis, esophagitis, psychotic disorder, and narcolepsy. A full list of the comorbidities reported is provided in the supplementary material as Table S1.

Table 1 also presents the key parameters of the sleep characteristics of the subjects obtained from the in-clinic PSG marking. On average, the total sleep time of the subjects was 453.6 min with a standard deviation of 42.0 min, whereas the sleep efficiency average was 82.6% with a standard deviation of 9.9%. The total sleep time of the subjects varied from 307.2 to 563.4 min with sleep efficiency ranging from 58.3% to 97.3%. The subjects on average were in the rapid eye movement (REM) stage for 15.5% of their sleep time with a median of 15.9% and a standard deviation of 6.8%. The percentage of sleep spent in the REM stage for the subjects ranged from 0% to 30%.

### 3.2. Diagnostic Accuracy

The diagnostic performance of AcuPebble SA100 using both AHI and ODI indices to identify moderate or severe OSA is shown in Table 2 and Table 3, respectively. This performance was obtained by comparing the diagnostic output from AcuPebble SA100 against equivalent diagnosis following the gold-standard method of in-clinic diagnosis using manually scored PSG.

**Figure 3:**
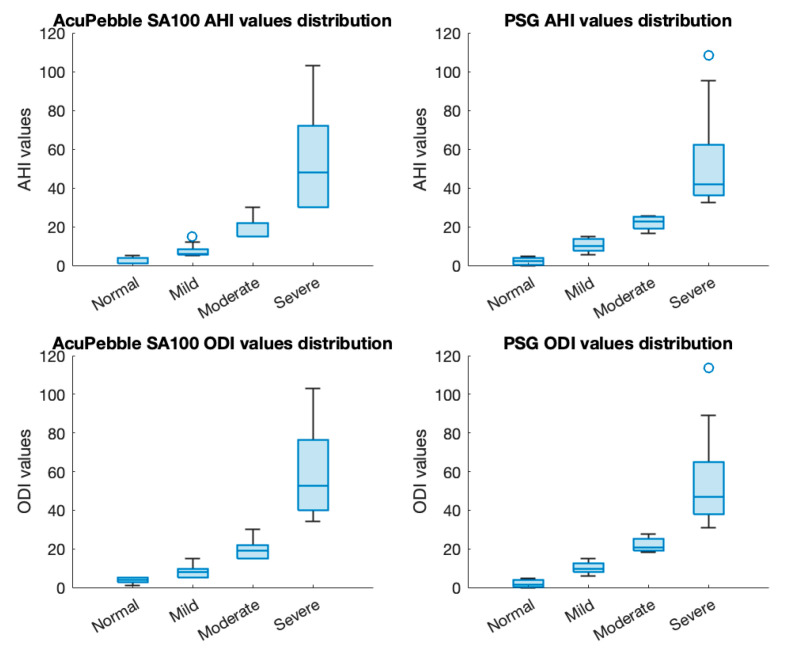
Distributions of AHI and ODI values of both AcuPebble SA100 and reference in-clinic PSG for different severity categories.

Table 2 presents the diagnostic performance of AcuPebble SA100 in identifying OSA using the AHI diagnostic index. It demonstrates an accuracy of 95.24%, with a sensitivity of 92.86% and a specificity of 97.14%. The PPV and NPV are 96.30% and 94.44%, whereas the LR+ and LR− are 32.50 and 0.07, respectively. Meanwhile, when utilizing the ODI as diagnostic criteria, AcuPebble SA100 achieves an overall accuracy of 92.06%, along with sensitivity and specificity values at 92.00% and 92.11%, PPV of 88.46%, NPV of 94.59%, LR+ of 11.65, and LR− of 0.09 (as shown in Table 3). Figure 3 presents the distributions of AHI and ODI values for both the output of AcuPebble SA100 and the gold-standard reference of in-clinic PSG for different severity categories.

AcuPebble SA100 is also capable of identifying apnea and hypopnea events and further classifying apneic events as central or obstructive. Its performance in identifying apnea and hypopnea events was evaluated by comparing its classification with that of a blind scorer for 10 patients with moderate or severe sleep apnea. The accuracy of this classification was found to be 89.77% (CI: 88.65% and 90.82%). When specifically classifying apnea events as central or obstructive, the accuracy was determined to be 82.54% (CI: 80.07%, 84.83%). These findings were validated using data from the aforementioned group of 10 patients who had the highest number of manually scored PSG-identified central events.

## 4. Discussion

Based on the results presented in this paper, the AcuPebble SA100 (EU) device has demonstrated an accurate automated diagnosis of OSA in patients undergoing in-clinic sleep testing when compared against the gold-standard reference PSG. For this study, the diagnostic outputs from AcuPebble SA100 were compared to the diagnosis the same patient would have received after having undergone in-clinic PSG, had the clinician followed the established diagnostic thresholds (i.e., 5, 15, 30) and the AASM recommendations for the definition of the events to determine the AHI, or alternatively if the clinician had opted for an ODI-based diagnosis with the same thresholds.

It is important to note that AcuPebble SA100 provides four automated diagnostic outputs to accommodate different global sleep centers’ criteria [15], but diagnoses based on AHI and ODI with 3% desaturations were chosen in this study following their recommendation in the AASM manual. The results showed high accuracy for both diagnostic criteria, as measured by various statistical metrics commonly used in assessing sleep apnea diagnosis methods. The accuracy was slightly higher when using AHI compared to ODI-based diagnoses, with an overall accuracy above 92% in both cases. It is also worth noting that, although somewhat expected, as this study only relies on diagnosis using AcuPebble SA100′s neck sensor, with no additional oximetry information from a conventional PPG-based oximeter, the algorithms are still able to detect decreases in oxygen saturation in OSA since this has characteristic cardio-respiratory acoustic features. The latter also results in the ability to extract an acoustic-derived desaturation channel that enables the identification of hypopneas based on reductions in oxygen saturation (rather than absolute values).

When using the AHI index, three studies were misclassified with 1 false positive and 2 false negatives. Using the ODI index, five studies were misclassified (3 false positives and 2 false negatives). In the case of the only false positive using the AHI, the reference diagnostic output had an AHI value of 14.3, very close to the positive diagnosis limit (15). This was also a false positive using the ODI index. In one of the other two cases of false positives with ODI, its value was 14.8, once again very close to the positive diagnosis limit. The third false positive had a larger discrepancy in ODI values (30 against a reference of 7.6). However, the AHI values in this test are consistent between both systems (21 and 25), which indicates a potential issue of poor finger sensor contact/perfusion in the reference system. In one of the false-negative cases, the reference diagnosis output had an AHI value of 18.1, while AcuPebble SA100′s output was 11. The other false negative had a larger discrepancy, not falling within the borderline range. Interestingly though it was found that a higher percentage of events identified by the reference were hypopnea in this specific case. However, it should be noted that even though these hypopnea events were marked in a post-comparison review, it was observed that they did not necessarily exhibit desaturation according to the output from the reference diagnostic system, and they were marked just based on the typical repetitive reduction of flow from the nasal pressure channel. However, the results presented here are completely blind, and hence, the tables have not been changed as a result of this post-comparison review. It is recognized that relying solely on nasal pressure channels may not provide sufficient correlation with actual airflow; hence considering factors such as square root transformation can enhance accuracy for event detection during sleep studies. Overall, these small differences between AcuPebble and PSG can be a consequence of several factors [15]. These include human error in event identification and rigorous duration assessment during manual scoring, as well as potential discrepancies caused by poor quality signals due to improper contact/sensor placement in PSG recordings. A larger study was previously reported with AcuPebble SA100 in its intended use environment (i.e., home) and replicating the real-world conditions (i.e., untrained patients using the device themselves), whilst using cardio-respiratory polygraphy with expert marking as a reference standard. Although this study is smaller, the results in terms of performance are similar to those obtained in the home environment situation. A side-by-side comparison of results is shown in Table 4. In both instances, the overall accuracy is just over 95%, and the other metrics exhibit comparable values well within the confidence interval ranges. The table also shows the performance results when the comparison from the two studies is pooled together.

Table 5 presents a comparative analysis of the diagnostic capabilities of AcuPebble SA100 in comparison to other automated sleep testing devices. The results indicate that AcuPebble SA100 demonstrates superior diagnostic performance. Specifically, when utilizing AHI as the diagnostic index, AcuPebble SA100 achieves a diagnostic sensitivity and specificity of 93.83% and 96.21%, respectively. This is better than those reported in [23] with NightOwl (91% and 76%), in [24] with Sunrise (100% and 75%), and in [25] with WatchPAT 200U (85.1% and 70.3%). It is essential to highlight that the performances reported in [24] are based on optimized thresholds for automatic diagnosis (indicated in red in Table 5), and the comparison index of the reference PSG differs from the traditional AHI used for OSA diagnosis.

As shown in the data sufficiency diagram (Figure 2), 17 patients could not be included in the comparison. The reasons for this are detailed before, but it is worth noting that out of those, seven patients had not slept a minimum of 240 m. Since AcuPebble sensors are very small and barely noticeable (as reported in [15]), this is representative of a typical percentage of patients who cannot sleep properly when attending an overnight in-hospital sleep study.

Although the results show a larger number of AcuPebble SA100 inconclusive tests (i.e., tests with non-diagnostic outputs) in the PSG than in the PG study, this is likely to be related to the fact that patients’ sleep position and quality are very different in a clinic whilst wearing many more wired sensors, than in their own bed with a reduced number. Since the algorithms have incorporated safety features to avoid giving a wrong diagnosis, some of these are conditional on factors, for example, the algorithms detecting signals that are not representative of intended use, and these features are likely to trigger more often if the patient is constrained than they would be at home. Hence, in this sense (i.e., percentage of inconclusive tests), we think the results from the home study are more representative than those obtained in PSG. Furthermore, it is also worth considering that since AcuPebble studies are very easy to repeat, considering the outputs are instantaneously available to clinicians, patients do not need to return to hospitals to be instructed to repeat the study, and the sensors are barely noticeable to patients, inconclusive studies pose an almost negligible burden for all.

The sample population in this study in isolation is smaller than the one in the previously reported validation study against cardiorespiratory polygraphy, plus expert marking, at home. However, this is somewhat justified by the fact that whilst PSG represents the gold standard, having patients in the hospital is less representative of what happens in the intended use situation for the device, i.e., intended to be used by patients with no training at home. It is also worth noticing that the smaller sample number is somewhat mirrored by the reality of sleep clinics, in which most of the patients are in fact not diagnosed with PSG by rather at home with either cardio-respiratory polygraph or simple oximetry. This study was carried out at the Virgen Macarena Hospital in Seville, Spain. The previous AcuPebble validation study [15] was carried out with patients enrolled at the Royal Free Hospital in London, UK. So, while the results of this particular study inherently demonstrate the performance of AcuPebble at a single center, the overall performance results of AcuPebble (as shown in Table 4) demonstrate its effectiveness across a very diverse population. When incorporated into diagnostic pathways, AcuPebble enables the streamlined diagnosis of OSA, addressing current bottlenecks in the process since, for a start, thanks to its ease of use, the device can be sent directly to patients without the need for any training from healthcare professionals. In addition, the ease of use of the device, together with the fact that it is reusable, makes it not just environmentally friendly but enables multi-night testing, which is a significant benefit compared to both the gold-standard and other systems since it allows to eliminate diagnostic uncertainties associated to night-to-night variability. Finally, as proven both in this paper and the previously presented home-based study [15], both the positive and likelihood ratios demonstrate that the automatic diagnostic output of the system can be trusted, without requiring any manual marking, to decide on subsequent steps in patient management. This not only cuts costs [15] but also expedites patients’ access to treatment pathways while also allowing reallocation of hospital resources. Future studies should focus on exactly quantifying how pathways could be optimized to maximize these benefits.

## 5. Conclusions

The results of this study demonstrate for the first time the performance of AcuPebble SA100 against PSG in a hospital environment. The device was previously validated against cardiorespiratory polygraphy (CR-PG) in the home environment, since this is the ultimate user case for it. This new study demonstrates very similar levels of diagnostic performance even if PSG is used as a reference, serving as additional evidence of the benefits a device such as AcuPebble SA100 could bring to the diagnostic pathways. The addition of a reliable automatic diagnosis, together with a cost-effective, easy-to-use, patient-friendly device, can result in time savings for human-resources-stretched diagnostic services, faster intervention decisions, facilitating diagnosis for vulnerable patients, and improved convenience for all. The ease of use and diagnostic reliability of the device could also enable its deployment to community clinics without necessitating specialized equipment or personnel, thereby increasing accessibility to sleep testing services. In summary, adopting emerging technologies with these characteristics could lead to cost savings within healthcare systems while significantly enhancing patients’ overall experience.

## Figures and Tables

**Table 1:** Demographic characteristic data of the patients.

Age (years)	Median	48
	Mean	50
	SD	12
	Range	(25, 78)
BMI	Median	28.7
	Mean	30.7
	SD	7.0
	Range	(16.7, 54.0)
Number of patients per BMI classification	Underweight (<18.5)	1 (1.59%)
	Healthy weight (18.5–24.9)	11 (17.46%)
	Overweight (25–29.9)	25 (39.68%)
	Obese (30–39.9) Severely obese (>40)	20 (31.75%) 6 (9.52%)
Sex	Male	40
	Female	23
Epworth Scale	Median	10
	Mean	10.4
	SD	6.8
	Range	(0, 24)
Stop Bang Score	Median	4
	Mean	4.1
	SD	1.6
	Range	(1, 8)
Total Sleep Time (minutes)	Median	456.7
	Mean	453.6
	SD	42.0
	Range	(307.2, 563.4)
Sleep Efficiency	Median	84.8%
	Mean	82.6%
	SD	9.9%
	Range	(58.3%, 97.3%)
Percentage of Sleep in REM	Median	15.9%
	Mean	15.5%
	SD	6.8%
	Range	(0%, 30%)

**Table 2:** Diagnostic performance of AcuPebble SA100 compared against the gold-standard reference of in-clinic PSG. A true positive (TP) corresponds to an AHI-based diagnosis of moderate or severe OSA.

Statistic	Value	95% CI
Sensitivity	92.86%	76.50% to 99.12%
Specificity	97.14%	85.08% to 99.93%
Positive predictive value	96.30%	78.98% to 99.45%
Negative predictive value	94.44%	81.71% to 98.48%
Positive likelihood ratio	32.50	4.70 to 224.92
Negative likelihood ratio	0.07	0.02 to 0.28
Disease prevalence	44.44%	-
Accuracy	95.24%	86.71% to 99.01%

**Table 3:** Diagnostic performance of AcuPebble SA100, where a true positive (TP) corresponds to an ODI-based diagnosis of moderate or severe OSA.

Statistic	Value	95% CI
Sensitivity	92.00%	73.97% to 99.02%
Specificity	92.11%	78.62% to 98.34%
Positive predictive value	88.46%	72.01% to 95.81%
Negative predictive value	94.59%	82.19% to 98.51%
Positive likelihood ratio	11.65	3.91 to 34.73
Negative likelihood ratio	0.09	0.02 to 0.33
Disease prevalence	39.68%	-
Accuracy	92.06%	82.44% to 97.37%

**Table 4:** Summary of validation performance for two different studies, the one reported in this paper (AcuPebble SA100 vs. PSC) and a former study [15] (AcuPebble vs. CR-PG at home).

		AcuP. vs. PSG in Clinic		AcuP. vs. CR-PG at Home		Combined Diagnostic Performance	
		CI		CI		CI	
AHI based validation	Sensitivity (%)	92.86	76.50 to 99.12	92.59	82.11 to 97.94	93.83	86.18 to 97.97
	Specificity (%)	97.14	85.08 to 99.93	96.88	91.14 to 99.35	96.21	91.38 to 98.76
	Positive Likelihood Ratio	32.50	4.70 to 224.92	29.63	9.70 to 90.48	24.77	10.47 to 58.63
	Negative Likelihood Ratio	0.07	0.02 to 0.28	0.08	0.03 to 0.20	0.06	0.03 to 0.15
	Disease prevalence (%)	44.44	31.92 to 57.51	36.00	28.33 to 44.23	38.03	31.48 to 44.91
	PPV (%)	96.30	78.98 to 99.45	94.34	84.52 to 98.07	93.83	86.53 to 97.30
	NPV (%)	94.44	81.71 to 98.48	95.88	90.05 to 98.35	96.21	91.57 to 98.34
	Accuracy (%)	95.24	86.71 to 99.01	95.33	90.62 to 98.10	95.31	91.54 to 97.73
ODI based validation	Sensitivity (%)	92.00	73.97 to 99.02	91.03	82.38 to 96.32	90.38	83.03 to 95.29
	Specificity (%)	92.11	78.62 to 98.34	93.06	84.53 to 97.71	93.58	87.22 to 97.38
	Positive Likelihood Ratio	11.65	3.91 to 34.73	13.11	5.61 to 30.62	14.07	6.85 to 28.90
	Negative Likelihood Ratio	0.09	0.02 to 0.33	0.10	0.05 to 0.20	0.10	0.06 to 0.19
	Disease prevalence (%)	39.68	27.57 to 52.80	52.00	43.70 to 60.22	48.83	41.94 to 55.75
	PPV (%)	88.46	72.01 to 95.81	93.42	85.87 to 97.07	93.07	86.74 to 96.50
	NPV (%)	94.59	82.19 to 98.51	90.54	82.48 to 95.11	91.07	84.96 to 94.85
	Accuracy (%)	92.06	82.44 to 97.37	92.00	86.44 to 95.80	92.02	87.53 to 95.28

**Table 5:** Diagnostic results comparison to other automated sleep testing devices.

	Device	Subjects Count	Index Test	Sensitivity % (95% CI)	Specificity % (95% CI)	PPV % (95% CI)	NPV % (95% CI)	Accuracy % (95% CI)	Tested at Intended Environment
This study	AcuPebble SA100	213	AHI ≥ 15 vs. AHI ≥ 15	93.83 (86.18–97.97)	96.21 (91.38–98.76)	93.83 (86.53–97.30)	96.21 (91.57–98.34)	95.31 (91.54–97.73)	Yes
		213	ODI ≥ 15 vs. ODI ≥ 15	90.38 (83.03–95.29)	93.58 (87.22–97.38)	93.07 (86.74–96.50)	91.07 (84.96–94.85)	92.02 (87.53–95.28)	
Van Pee (2022) [23]	NightOwl	228	PAT-AHI ≥ 15 vs. PSG-AHI ≥ 15	91 (85–96)	76 (65–87)	82 (72–92)	88 (81–94)	86 (80–91)	No
Kelly (2022) [24]	Sunrise	31	MM-ORDI > 12.65 vs. PSG ORDI > 15	100 (79–100)	75 (45–92)	80	100	88	No
Pillar (2020) [25]	WatchPAT 200U	84	WP-AHI ≥ 15 vs. PSG AHI ≥ 15	85.1	70.3	79.4	78.8	86	No

## Data Availability

Data are available upon reasonable request. Data of individual participants will not be shared since consent for this has only been granted to regulatory authorities. All other information will be shared on request and provided it is not confidential for IP protection reasons. Requests should be directed to: e.rodriguez@imperial.ac.uk.

## Supplementary Materials

The following supporting information can be downloaded at: https://www.mdpi.com/article/10.3390/jcm13020571/s1, Table S1: List of comorbidities for subjects included in the analysis.
