# Supplementary material for: Validation of a Wearable Medical Device for Automatic Diagnosis of OSA against Standard PSG
Source: J Clin Med. 2024 Jan 19;13(2):571. doi: 10.3390/jcm13020571 (PMC10816319; doi:10.3390/jcm13020571)
Supplement: Supplementary file 1 [file jcm-13-00571-s001.zip › jcm-2801427-supplementary.pdf]

Table S1 - List of comorbidities for subjects included in the analysis

Comorbidities	Subjects count
Nocturia	25
Hypertension	17
Obesity	9
Cardiopathies	7
Diabetes	6
Hypothyroidism	4
Depressive Anxiety Disorder	3
Asthma	3
Dyslipidemia	2
Sleep Maintenance Insomnia	2
Epilepsy	2
Turbinoplasty	1
Floppy Eyelid Syndrome	1
Irritable Colon	1
Megaloblastic Anemia	1
Lumbocoatalgia	1
Sleep Onset Insomnia	1
Allergic Rhinitis	1
Multiple Sclerosis	1
Posterior Territory Ischaemic Stroke	1
HIV	1
Bronchial hyper-reactivity	1
Depressive Disorder	1
Crohn's Disease	1
Turbinate Hypertrophy	1
Chronic Diarrhea	1
Gilbert's Syndrome	1
Scoliosis	1
Fibromyalgia	1
Bruxism	1
Sleepwalking	1
Diabetic Neuropathy	1
Multinodular Goiter	1
Psoriatic Arthritis	1
Esophagitis	1

Psychotic Disorder	1
Narcolepsy	1
